# A Model Combining Skeletal Muscle Mass and a Hematological Biomarker to Predict Survival in Patients With Nasopharyngeal Carcinoma Undergoing Concurrent Chemoradiotherapy

**DOI:** 10.3389/fonc.2021.644676

**Published:** 2021-05-18

**Authors:** Han-Ying Huang, Fei Lin, Xiao-Yu Chen, Wen Wen, Shuang-Yan Xie, Zhi-Qing Long, Ling Guo, Huan-Xin Lin

**Affiliations:** ^1^ State Key Laboratory of Oncology in South China, Guangdong Key Laboratory of Nasopharyngeal Carcinoma Diagnosis and Therapy, Sun Yat-sen University Cancer Center, Collaborative Innovation Center for Cancer Medicine, Guangzhou, China; ^2^ Department of Radiotherapy, Sun Yat-sen University Cancer Center, Guangzhou, China; ^3^ Department of Nasopharyngeal Carcinoma, Sun Yat-sen University Cancer Center, Guangzhou, China

**Keywords:** nasopharyngeal carcinoma, sarcopenia, tumor lymph node metastasis, nomogram, survival

## Abstract

**Background:**

Using the current tumor lymph node metastasis (TNM) staging system to make treatment decisions and predict survival in patients with nasopharyngeal carcinoma (NPC) lacks sufficient accuracy. Patients at the same stage often have different survival prognoses.

**Methods:**

In the current study 802 NPC patients who underwent concurrent radiotherapy and chemotherapy from January 2010 to December 2014 at Sun Yat-sen University Cancer Center in China were retrospectively assessed. The optimal cut-off points for skeletal muscle index (SMI) and monocyte-to-lymphocyte ratio (MLR) were determined *via* receiver operating characteristic curves. SMI-MLR (S-M) grade and a nomogram were developed and used as clinical indicators in NPC patients. The consistency index (C-index) and a calibration curve were used to measure the accuracy and discriminative capacity of prediction.

**Results:**

The predictive performance of S-M grade was better than that of TNM staging (C-index 0.639, range 0.578–0.701 *vs.* 0.605, range 0.545–0.665; *p* = 0.037). In multivariate analysis S-M grade, T stage, and N stage were independent prognostic factors. These three factors were then combined, yielding a nomogram with a C-index of 0.71 (range 0.64–0.77), indicating good predictive capacity.

**Conclusion:**

We developed and validated a prognostic parameter, S-M grade, which increased prediction accuracy significantly and can be combined with TNM staging to predict survival in patients with NPC undergoing concurrent chemoradiotherapy.

## Introduction

Nasopharyngeal carcinoma (NPC) is a common malignant tumor in southern China. It differs greatly by ethnicity and geographic distribution, and exhibits diverse histopathology (1–3). At the time of initial diagnosis more than 70% of NPC patients are locally advanced (4). Radiotherapy and chemotherapy based on the tumor lymph node metastasis (TNM) staging system are currently recognized as the main treatment methods for locally advanced NPC (5, 6). Unfortunately, patients with NPCs of the same stage often have different prognoses (7).

To provide the optimal treatment to the patient at the right time, precision medicine has been emphasized for the treatment of disease based on individual differences. Though the TNM staging system is the most widely used to predict prognosis and guide treatment, it only grades patients based on the degree of lymph node involvement and the size of the tumor, and ignores other important prognostic factors such as nutritional features, treatment-related factors, and inflammation status. It has been proposed that individual prediction of survival could be optimized by taking these factors into account (8–10).

Sarcopenia is evidently an informative prognostic factor that can affect treatment efficacy and survival in patients with head and neck cancer (11–13). Studies indicate that sarcopenia evaluated *via* the skeletal muscle index (SMI) may also be related to responses to concurrent chemoradiotherapy (CCRT), and its toxicity (14, 15). The monocyte-to-lymphocyte ratio (MLR) in the blood is negatively correlated with overall survival in NPC patients (16), and it may be a useful addition to TNM staging in the prognostic assessment of NPC patients (17). Notably however, there is no report of the combined use of these indicators to construct a model to predict prognoses in NPC patients undergoing CCRT. To facilitate more informed clinical decision-making, a comprehensive and convenient tool combining SMI, MLR, and TNM stage could be developed.

The aim of the present study was to construct a practical tool combining SMI, MLR, and TNM stage to assess the prognosis of patients with NPC undergoing CCRT.

## Methods

### Patients

A total of 802 NPC patients who had undergone CCRT from January 2010 to December 2014 at Sun Yat-sen University Cancer Center, Guangzhou, China, were retrospectively enrolled in the study. Data including age, sex, histological type, monocyte count, lymphocyte count, TNM stage, Epstein‑Barr virus DNA test results, and skeletal muscle index were analyzed. The inclusion criteria were (a) confirmed non-metastatic NPC, (b) serological testing and radiographic examination prior to treatment, and (c) cisplatin 100 mg/m^2^ every 3 weeks and CCRT with radical intensity-modulated radiotherapy conducted in accordance with the guidelines of our institute (18). The study was approved by the Research Ethics Committee of Sun Yat-sen University Cancer Center (approval number GZR2017-224). All patients provided written informed consent for the treatment, and agreed to cooperate with follow-up requirements.

### Data Collection and Definitions

The SMI was used to evaluate the presence of sarcopenia, and was calculated as the patient’s skeletal muscle area (cm^2^) divided by their height squared (m^2^). The method described in Hua et al. (19) was used to measure skeletal muscle area at the third cervical vertebrae level. Routine clinicopathological data were collected within 1 week of treatment, and the monocyte count divided by the lymphocyte count was used as the MLR. The optimal cutoff points for SMI and MLR were determined *via* receiver operating characteristic (ROC) curves. The patients were randomly divided into a training set of 561 and a validation set of 241 to construct a nomogram.

### CCRT Treatment and Follow-Up

The patients received 100 mg/m^2^ cisplatin every 3 weeks concurrently with radical intensity‑modulated radiotherapy administered in accordance with the guidelines of our institute (18). Three months after CCRT, nasopharyngoscopic examination and magnetic resonance imaging were performed in accordance with RECIST 1.1 standards to assess treatment responses (20). The patients were followed up *via* outpatient examinations or telephone interviews. Overall survival (OS) was defined as the time from the date of diagnosis to the date of death or the date of the last follow-up.

### Statistical Analysis

Statistical analyses were conducted using SPSS version 23.0 (IBM Corp., Armonk, New York, USA), GraphPad Prism version 6.0 software (GraphPad, La Jolla, California, USA), and R software version 3.6.2 (R Foundation for Statistical Computing, Vienna, Austria). ROC curve analyses were used to determine optimal cutoff points for the SMI and MLR. Survival curves were calculated using the Kaplan–Meier method and compared using the log-rank test. Univariate and multivariate analyses were performed using a Cox proportional hazards model, and the multivariate model included variables with *p* values < 0.20 in the univariate analysis. *p <*0.05 was deemed to indicate that the Cox proportional hazards regression assumption was violated. A nomogram for the combined model including S-M grade, N stage, and T stage was generated. A calibration curve was used for self‑verification of the nomogram. Two-tailed *p* values < 0.05 were considered statistically significant.

## Results

### Definitions and Baseline Co-Variates

ROC curves indicated that the optimal cut-off points for sarcopenia were SMI < 21.99 cm^2^/m^2^ (male) and < 18.60 cm^2^/m^2^ (female), and the optimal cut-off point for MLR was 0.22. Based on these cut‑offs, S-M grades were defined as follows: Grade 1, non-sarcopenia + low MLR; grade 2, non‑sarcopenia + high MLR/sarcopenia + low MLR; grade 3, sarcopenia + high MLR. The characteristics of the 802 patients retrospectively enrolled in the study are shown in **Table 1**. Their median age at diagnosis was 45 years (range 18–84 years), their median SMI was 24.52 cm^2^/m^2^ (range 10.96–57.46 cm^2^/m^2^), and their median MLR was 0.21 (range 0.01–2.39).

**Table 1:** Patient demographics and clinical characteristics.

Characteristic	Number of patients (%)
**Age**	
≥45 years	390(48.6)
<45 years	412(51.4)
**Gender**	
Male	598(74.6)
Female	204(25.4)
**Histological type**	
WHO I	4(0.5)
WHO II	9(1.1)
WHO III	789(98.4)
**MLR**	
≥0.22	363(45.3)
<0.22	439(54.7)
**T stage**	
T1	39(4.9)
T2	154(19.2)
T3	493(61.5)
T4	116(14.5)
**N stage**	
N0	76(9.5)
N1	432(53.9)
N2	251(31.3)
N3	43(5.4)
**TNM stage**	
II	113(14.1)
III	536(66.8)
IV	153(19.1)
**EBV-DNA**	
<4000 copy/mL	530(66.1)
≥4000 copy/mL	272(33.9)
**Sarcopenia**	
No	605(75.4)
Yes	197(24.6)
**S-M grade**	
grade 1	325(40.5)
grade 2	394(49.1)
grade 3	83(10.3)

WHO, World Health Organization; MLR, monocyte-to-lymphocyte ratio; SMI, skeletal muscle index; EBV-DNA, Epstein-Barr virus DNA; S-M grade, SMI-MLR grade.

### Comparisons of S-M Grade and TNM Stage

**Figure 1:**
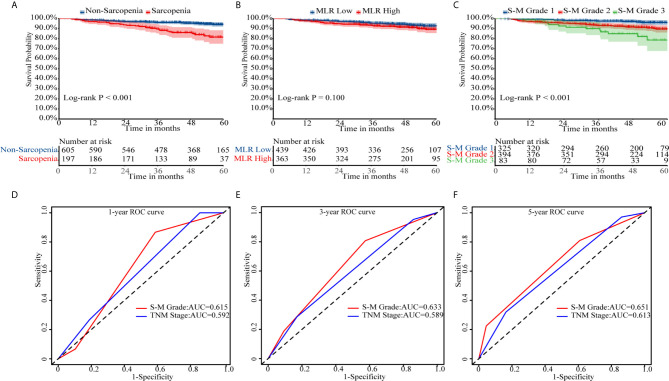
Kaplan–Meier survival curves and receiver operating characteristic curve analysis. Kaplan–Meier curves for overall survival by **(A)** skeletal muscle index, **(B)** monocyte-to-lymphocyte ratio, and **(C)** combined skeletal muscle index and monocyte-lymphocyte ratio (S-M) grade. **(D–F)** Capacities of S-M grade and TNM stage to predict overall survival for 1, 3, and 5 years. S-M grade, combined skeletal muscle index and monocyte-lymphocyte ratio.

In Kaplan–Meier survival analysis, survival differed depending on whether sarcopenia (*p* < 0.001, **Figure 1A**) or MLR (*p* = 0.100, **Figure 1B**) was used. Patients with S-M grade 1 had higher OS than patients with S-M grades 2 or 3 (*p* < 0.001, **Figure 1C**). The patient’s survival rate was significantly different with the TNM stage (*p* = 0.0018, **Supplementary Figure 4**). The respective C-indexes for S-M grade, TNM stage, SMI, and MLR were 0.639 (0.578–0.701), 0.605 (0.545–0.665), 0.622 (0.557–0.687), and 0.554 (0.489–0.619) (**Table 2**). Using S-M grade resulted in significantly more accurate prognostic prediction than using TNM stage (*p* = 0.037). In time-dependent ROC analyses for survival at 1, 3, and 5 years, S-M grade and TNM stage yielded similar results. The area under the curve values at 1, 3, and 5 years were higher for S-M grade than for TNM stage (**Figures 1D–F**). S-M grade predicted OS outcomes more accurately than TNM stage, but there was no significant difference between the two with respect to disease-free survival or relapse-free survival (**Supplementary Figure 1**).

**Table 2:** The C-index of S-M grade, TNM stage, SMI, and MLR for prediction of overall survival.

Characteristic	C-index (95%CI)	P
S-M grade	0.639(0.578-0.701)	
TNM stage	0.605(0.545-0.665)	
SMI	0.622(0.557-0.687)	
MLR	0.554(0.489-0.619)	
S-M grade *vs.* TNM stage		0.037
S-M grade *vs.* SMI		0.056
S-M grade *vs.* MLR		0.002

P values are calculated based on normal approximation using function rcorrp. cens in Hmisc package.

C-index, concordance index; CI, confidence interval; MLR, monocyte-to-lymphocyte ratio; SMI, skeletal muscle index; S-M grade, SMI-MLR grade.

### Univariate and Multivariate Analyses

The estimated hazard ratio for each prognostic factor is only valid if the underlying proportional hazards regression hypothesis is true. The Schoenfeld residuals over time were drawn proportionally for each prognostic factor included in the multivariate analysis (**Supplementary Figure 2**). The six variables—age, historical type, T stage, N stage, Epstein-Barr virus DNA test results, and S-M grade—were not statistically significant, indicating that the proportional hazards regression hypothesis was true over time. The results of univariate and multivariate analyses of prognostic indicators of OS are shown in **Table 3**. In univariate analysis T stage, N stage, Epstein-Barr virus DNA titer, and S-M grade were significantly correlated with OS (*p* < 0.05). In multivariate analyses including variables with *p* values < 0.20 in the univariate analysis, T stage, N stage, and S-M grade were independent prognostic factors.

**Table 3:** Univariate and multivariate analyses of overall survival.

Characteristics	Univariate analysis		Multivariate analysis	
	Hazard ratio(95%CI)	P	Hazard ratio(95%CI)	P
**Age**				
≥45 years	1		1	
<45 years	0.657(0.395-1.093)	0.106	0.666(0.399-1.114)	0.122
**Gender**				
Male	1			
Female	0.795(0.431-1.469)	0.464		
**Histological type**				
WHO III	1		1	
WHO I/ II	3.035(0.948-9.720)	0.062	2.816(0.850-9.330)	0.090
**T stage**				
T1-2	1		1	
T3	1.613(0.778-3.342)	0.199	1.608(0.767-3.370)	0.209
T4	2.971(1.299-6.793)	0.010	2.685(1.145-6.298)	0.023
**N stage**				
N0-1	1		1	
N2	2.214(1.303-3.762)	0.003	2.269(1.314-3.918)	0.003
N3	3.000(1.235-7.283)	0.015	2.594(1.042-6.463)	0.041
**EBV-DNA**				
<4000 copy/mL	1		1	
≥4000 copy/mL	1.942(1.173-3.214)	0.010	1.448(0.853-2.459)	0.171
**S-M grade**				
grade 1	1		1	
grade 2	2.538(1.320-4.878)	0.005	2.450(1.268-4.733)	0.008
grade 3	4.839(2.205-10.618)	<0.001	3.884(1.747-8.635)	0.001

Hazard ratios estimated by Cox proportional hazards regression. All statistical tests were two-sided.

CI, confidence interval; HR, hazard ratio; WHO, World Health Organization; EBV-DNA, Epstein-Barr virus DNA; MLR, monocyte-to-lymphocyte ratio; SMI, skeletal muscle index; S-M grade, SMI-MLR grade.

### Constructing a Combined Nomogram

**Figure 2:**
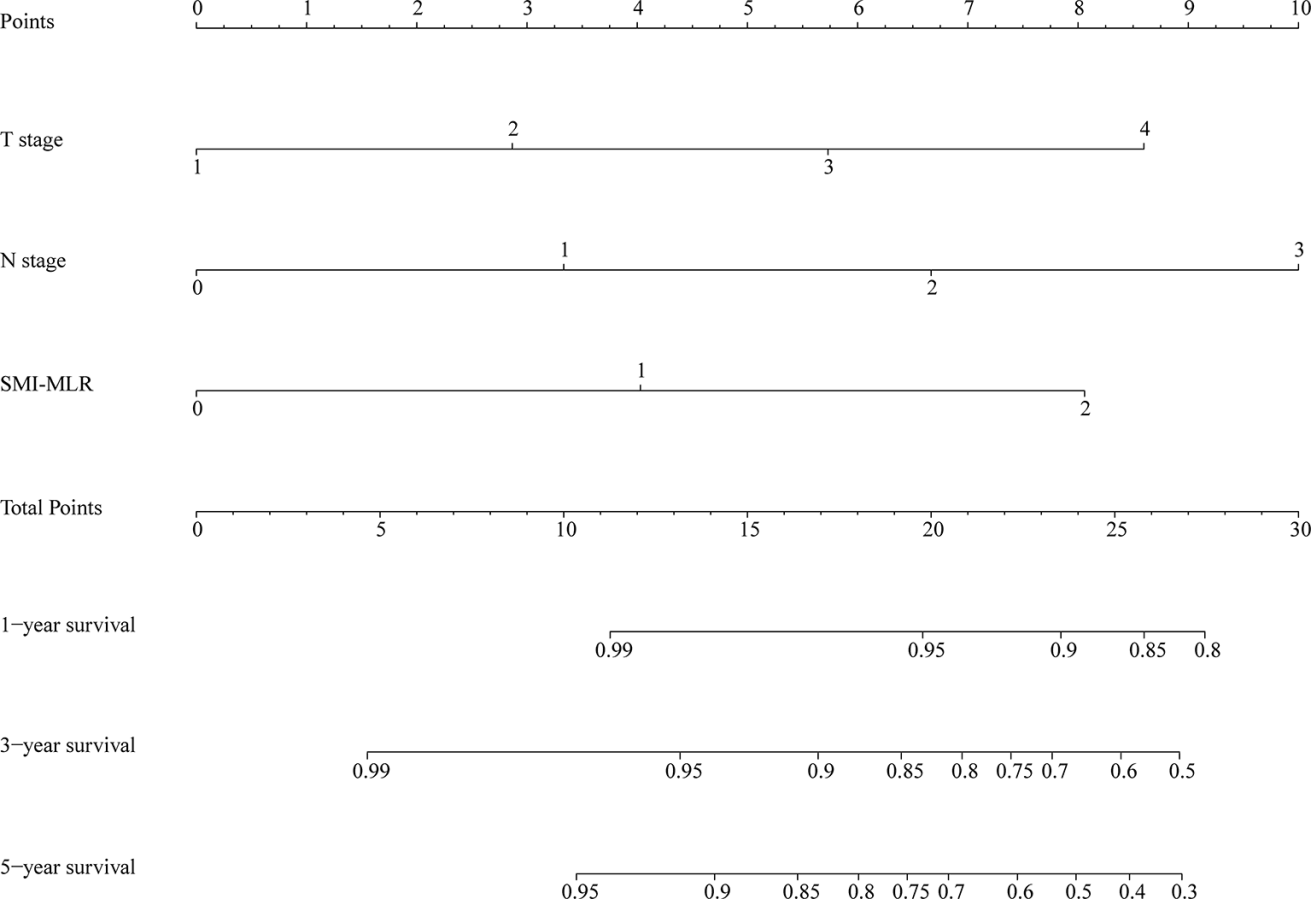
Nomogram to predict 1-, 3-, and 5-year overall survival generated using the training set.

**Figure 3:**
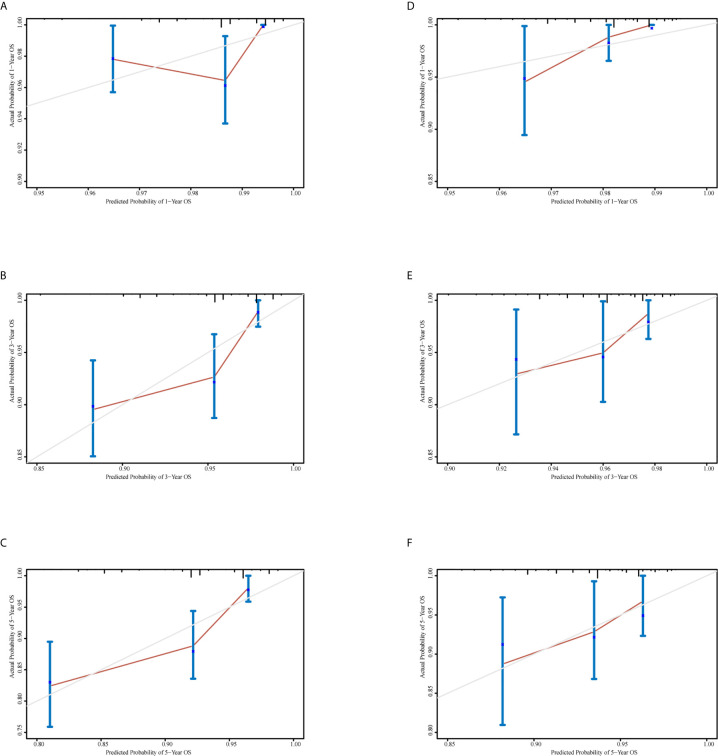
Calibration curves to predict 1-, 3-, and 5-year overall survival in the training set and the validation set. **(A–C)** Calibration curves for 1-, 3-, and 5-year overall survival in the training set. **(D–F)** Calibration curves for 1-, 3-, and 5-year overall survival in the validation set.

A nomogram was generated using a random sample of 70% of the cases (*n* = 561) as a training set, and the remaining 30% of cases (*n* = 241) were used to verify the model. The nomogram included S‑M grade, N stage, and T stage and was used to predict 1, 3, and 5-year OS (**Figure 2**). The C‑indexes of the nomogram were 0.74 (0.67–0.82) in the training set and 0.68 (0.52–0.84) in the validation set. In the graphs shown in **Figure 3** the x-axes represent nomogram-calculated predicted survival, the y-axes represent Kaplan–Meier-estimated observed survival, and the solid lines represent the ideal reference line for which actual survival and predicted survival correspond. The calibration plot for the probability of post-treatment 1, 3, and 5-year OS indicated optimal agreement between the prediction by the nomogram and actual observation for the nomogram. Compared with TNM staging determined by a time-dependent ROC curve, the area under the ROC curve of the nomogram was always higher (**Supplementary Figure 3**). The comprehensive predictive capacity of the nomogram was better than that of TNM.

## Discussion

The American Joint Committee on Cancer TNM staging system is used as the benchmark to predict prognosis and guide treatment in NPC, but it is somewhat controversial (21). For example, it is only based on the anatomical extent of the disease and does not completely account for the biological heterogeneity of patients with NPC. In addition, the system does not incorporate other risk factors. In patients with NPC, predictive accuracy could be affected by these issues.

Low SMI, termed sarcopenia, often occurs in head and neck cancer and is associated with reduced survival in patients with NPC. SMI can be conveniently determined using computed tomography simulation scans, which are part of pre-treatment evaluation prior to radiotherapy. MLR is also used widely in clinical applications, and it is believed that its utilization in conjunction with the TNM staging system can be useful. The combination of TNM staging, SMI, and MLR may substantially improve individualized predictions of survival, but to date no effective method has been developed to combine MLR and SMI to predict prognosis and guide treatment.

In the past, the SMI and MLR have been reported as single factors in NPC. A previous study indicated that the composition of human tissues such as visceral adipose tissues was related to patient prognoses and immune responses (22). Skeletal muscle loss in NPC patients during radiotherapy and chemotherapy may shorten a patient’s OS (23). In a meta-analysis it was concluded that sarcopenia before treatment had a significant negative effect in patients with head and neck tumors (24). In another meta-analysis low MLR predicted significantly more favorable OS (25). Although the correlation between MLR and prognosis was not statistically significant in the current study, low MLR was associated with better OS, which is consistent with the aforementioned studies. Notably however, hitherto the combination of SMI and MLR for NPC has not been reported. Combining the two indicators can better guide treatment and prognosis.

In the present study, a new model was developed based on a constructed parameter termed the S-M grade, which reflects the SMI and MLR combined, and it was used to predict OS in patients with NPC undergoing CCRT. The capacity of S-M grade to predict OS was compared with that of the TNM staging system, and was found to be more accurate. S-M grade was an independent prognostic factor. A new nomogram was developed that incorporates N stage, T stage, and S-M grade, and it exhibited greater predictive accuracy than the conventional TNM staging system. The C-index and area under the curve values of S-M grade were higher than those of the current staging system, as was the C‑index of the novel nomogram, indicating that the above-described concerns were addressed by the new method.

According to reports, CCRT improves the prognosis of stage II-IV NPC patients, and the 5-year OS rate of patients in different centers and stages ranges from 68% to 94.5% (26–28). In our center, the 1-year, 3-year, and 5-year OS rates of TNM were 98.1% ± 0.98%, 94.4% ± 1.57%, and 91.1% ± 2.35%, respectively. The 5-year OS rate of TNM stage II, stage III, and stage IV were 98.1% ± 2.55%, 91.5% ± 2.74%, and 84.2% ± 6.66%, respectively. The OS rate of our patients was relatively high, which may be due to early treatment and compliance, daily care, and rehabilitation in our center.

In addition, many models were currently being explored and tried to be combined with TNM to improve the performance of patient prognosis prediction. Zhu et al. (28) combined the nomogram of age, T stage, N stage, peripheral neutrophil–lymphocyte ratio, and lactic dehydrogenase to improve the predictive performance, but the number of patients was small. Zheng et al. (29) study prognostic nomograms based on 9 biomarkers, but these indicators were not routinely measured before treatment, so they are not suitable for wide application. Xiang et al. (9) established a new prognostic model based on C-reactive protein and N classification to predict the rate of distant metastasis without validation. Xie et al. (30) developed a nomogram based on the UICC 2002 TNM staging system to predict the prognosis of NPC patients undergoing IMRT with or without chemotherapy. However, there were only 44 patients with concurrent radiotherapy and chemotherapy in the verification cohort, which resulted in a prognostic bias.

The new and easy-to-use scoring system described herein can be utilized by clinicians to perform post-treatment individualized survival prediction. Treatment and care options could be tailored to fit subgroups of patients with different risks of poor survival identified *via* the new method. There is controversy surrounding the identification of patients who require intensive follow-up or additional therapy, and this could be resolved using our novel scoring system. Moreover, the tool developed could produce information that was useful for patient stratification when designing clinical studies, which may result in better equivalence between study arms.

The current study had several limitations. It only included Chinese patients at a single Cancer Center, and most of them resided in southern China. Thus, the prediction model is not applicable to all people. Another limitation was the retrospective nature of the study, which may have resulted in a degree of selection bias. It was worth noting that the training cohort and the validation cohort came from a unified medical record, and their baseline level was uniform, which maked the two cohorts comparable to the baseline. Lastly, the number of NPC patients in the study was comparatively small. To solve these problems a randomized controlled trial with a larger sample size is required, to improve the model and optimize it for clinical practice. In the future we may conduct a randomized, controlled, multi-center, international collaborative trial in an effort to achieve in-depth verification of the stability and practicability of the model.

Our model did not include another potentially valuable prognostic factor (EBV DNA). This is a limitation that should be considered. Many studies have confirmed that plasma EBV DNA is an important prognostic marker (31). However, the study of Zeng et al. (32) showed that there was no significant difference in the C index of models with or without EBV-DNA in the validation set (*p* = 0.09). Moreover, most centers cannot routinely perform EBV DNA testing, so many patients do not have relevant data. Quantitative plasma EBV DNA testing has not been standardized globally, and the results of different laboratories have produced huge differences in copy number. Standardization of EBV DNA serological testing is needed to make the results comparable in different research studies.

In conclusion, in the present study we developed and validated a prognostic model for predicting survival in NPC patients undergoing CCRT that was more precise than the TNM staging system. S-M grade, which was an independent predictor of survival in multivariate analyses, increased prediction accuracy significantly and can be combined with TNM staging to predict survival in patients with NPC.

## Data Availability Statement

The original contributions presented in the study are included in the article/**Supplementary Material**. Further inquiries can be directed to the corresponding authors.

## Ethics Statement

In the present study, all the procedures involving human participants were performed according to the ethical standards of the institutional research committee or the national research committee, or both, and with the 1964 Helsinki declaration and its later amendments or comparable ethical standards. The Clinical Research Ethics Committee of SYSUCC approved this study (number: GZR2017-224). All individual participants in the study provided informed consent.

## Author Contributions

Conceptualization: H-YH, H-XL, and LG. Data curation: FL, X-YC, and WW. Formal analysis: H-YH. Funding acquisition: H-XL and LG. Investigation: FL, X-YC, and Z-QL. Methodology: H-YH. Project administration: H-XL and LG. Software: H-YH, FL, and X-YC. Supervision: H-XL and LG. Validation: H-YH, FL, and X-YC. Visualization: H-YH. Writing—original draft: H-YH. Writing—review and editing: all authors. All authors contributed to the article and approved the submitted version.

## Funding

H-XL is supported by the National Natural Science Foundation of China (grant 81773103) and the Natural Science Foundation of Guangdong Province (2017A030313617). LG is supported by the National Natural Science Foundation of China (grant 81772877 and 81572848).

## Conflict of Interest

The authors declare that the research was conducted in the absence of any commercial or financial relationships that could be construed as a potential conflict of interest.

## Supplementary Material

Supplementary Figure 1 Kaplan–Meier curves for disease-free survival **(A)** and relapse-free survival **(B)**. DFS, disease-free survival; RFS, relapse-free survival.

Supplementary Figure 2 A scaled Schoenfeld residual plot for age **(A)**, histological type **(B)**, T stage **(C)**, N stage **(D)**, Epstein-Barr virus DNA **(E)**, and combined skeletal muscle index and monocyte-lymphocyte ratio grade **(F)**. PH, proportional hazard.

Supplementary Figure 3 Comparison of the capacities of the nomogram and tumor lymph node metastasis (TNM) stage by time-dependent receiver operating characteristic curve to predict overall survival. The red line represents the nomogram, and the black line represents the TNM stage. The dashed line is the 95% confidence interval.

Supplementary Figure 4 Kaplan–Meier curves for overall survival by TNM.
