# Supplementary figures and images for: A Model Combining Skeletal Muscle Mass and a Hematological Biomarker to Predict Survival in Patients With Nasopharyngeal Carcinoma Undergoing Concurrent Chemoradiotherapy
Source: Front Oncol. 2021 May 18;11:644676. doi: 10.3389/fonc.2021.644676 (PMC8167045; doi:10.3389/fonc.2021.644676)

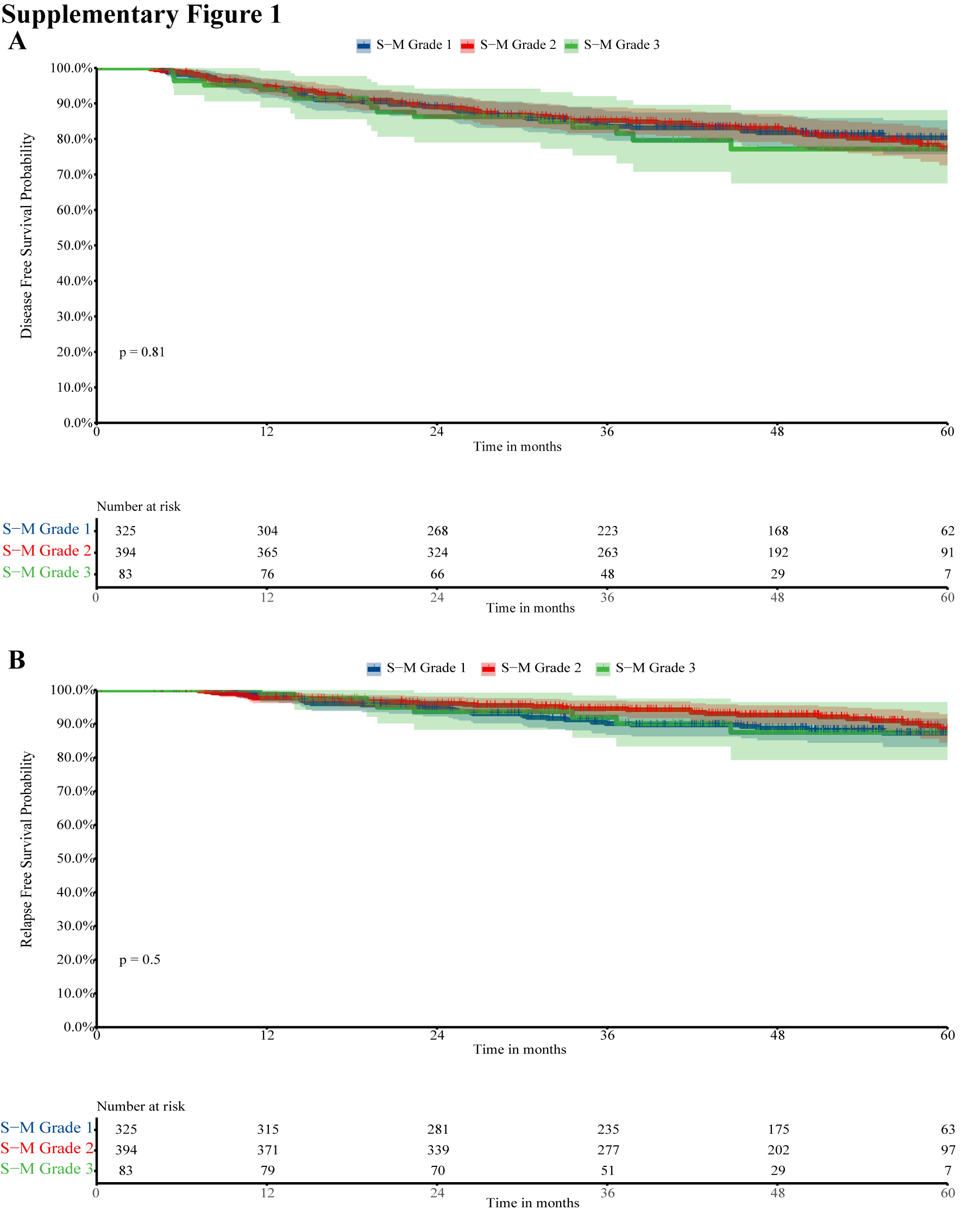

Supplement: Supplementary Figure 1 — Kaplan–Meier curves for disease-free survival (A) and relapse-free survival (B). DFS, disease-free survival; RFS, relapse-free survival. [file Image_1.tif]

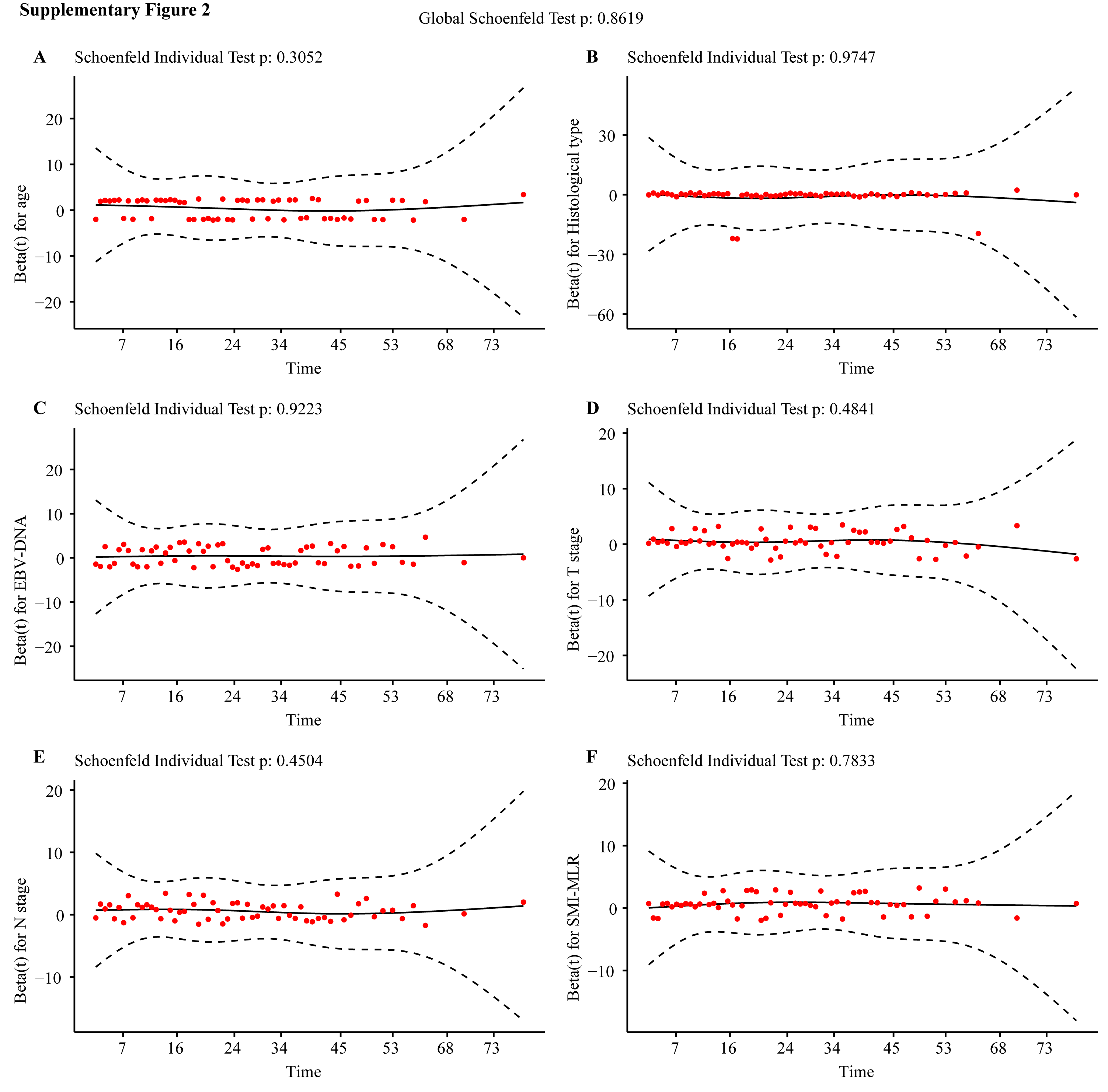

Supplement: Supplementary Figure 2 — A scaled Schoenfeld residual plot for age (A), histological type (B), T stage (C), N stage (D), Epstein-Barr virus DNA (E), and combined skeletal muscle index and monocyte-lymphocyte ratio grade (F). PH, proportional hazard. [file Image_2.tif]

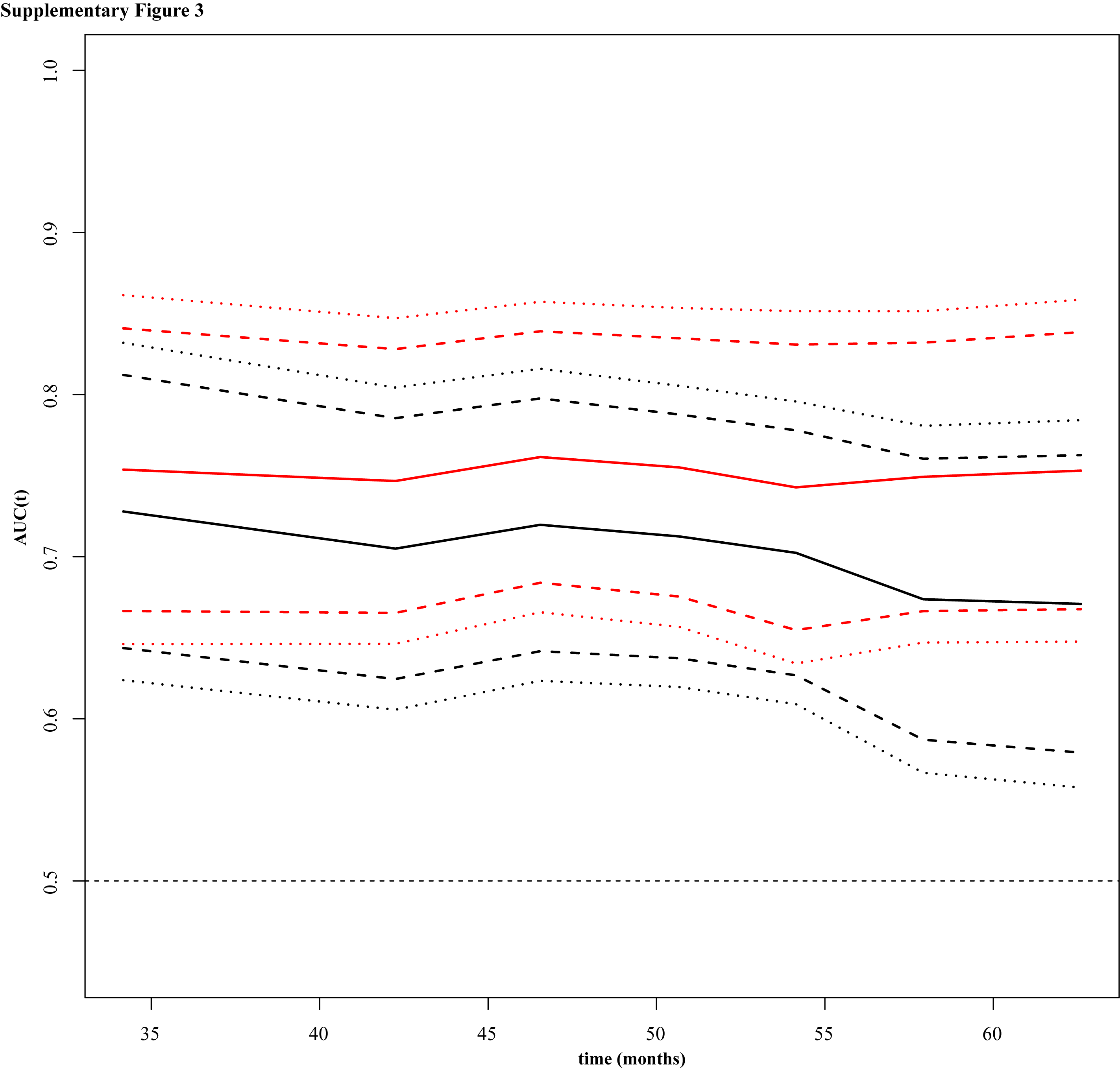

Supplement: Supplementary Figure 3 — Comparison of the capacities of the nomogram and tumor lymph node metastasis (TNM) stage by time-dependent receiver operating characteristic curve to predict overall survival. The red line represents the nomogram, and the black line represents the TNM stage. The dashed line is the 95% confidence interval. [file Image_3.tif]

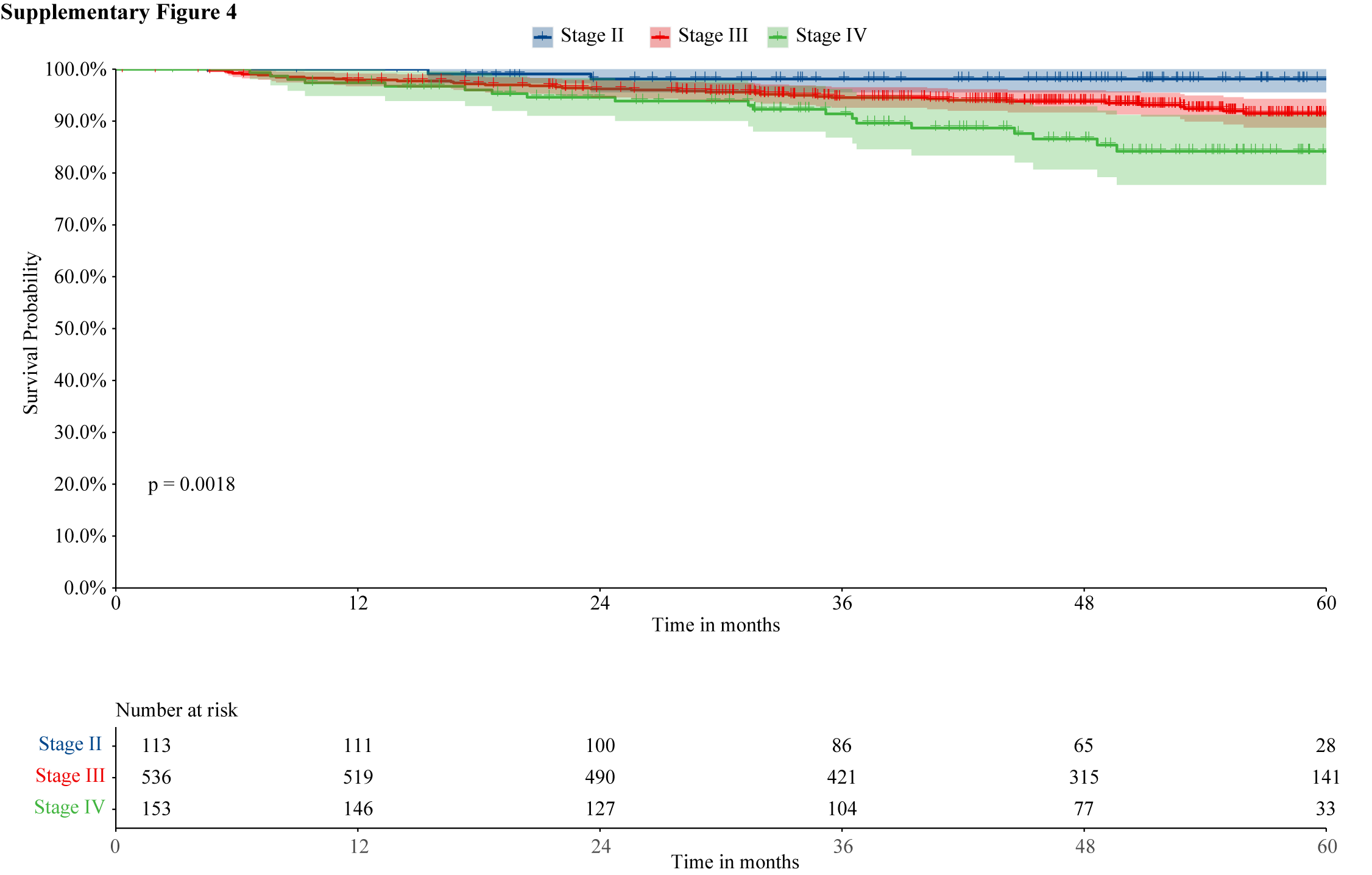

Supplement: Supplementary Figure 4 — Kaplan–Meier curves for overall survival by TNM. [file Image_4.tif]
